# Non-Mammalian Models for Understanding Neurological Defects in RASopathies

**DOI:** 10.3390/biomedicines12040841

**Published:** 2024-04-10

**Authors:** Mario Rodríguez-Martín, Juan Báez-Flores, Vanessa Ribes, María Isidoro-García, Jesus Lacal, Pablo Prieto-Matos

**Affiliations:** 1 Laboratory of Functional Genetics of Rare Diseases, Department of Microbiology and Genetics, University of Salamanca, 37007 Salamanca, Spain; mario.rm@usal.es (M.R.-M.); alumni.jbaez@usal.es (J.B.-F.); 2 Institute of Biomedical Research of Salamanca (IBSAL), 37007 Salamanca, Spain; misidoro@saludcastillayleon.es (M.I.-G.); pprieto@saludcastillayleon.es (P.P.-M.); 3 Institut Jacques Monod, Université Paris Cité, CNRS, F-75013 Paris, France; vanessa.ribes@ijm.fr; 4 Clinical Biochemistry Department, Hospital Universitario de Salamanca, 37007 Salamanca, Spain; 5 Clinical Rare Diseases Reference Unit DiERCyL, 37007 Castilla y León, Spain; 6 Department of Medicine, University of Salamanca, 37007 Salamanca, Spain; 7 Department of Pediatrics, Hospital Universitario de Salamanca, 37007 Salamanca, Spain; 8 Department of Biomedical and Diagnostics Science, University of Salamanca, 37007 Salamanca, Spain

**Keywords:** RASopathies, neurodevelopmental disorders, RAS/MAPK pathway, non-mammalian models, neurobiology, phenotype–genotype relationships

## Abstract

RASopathies, a group of neurodevelopmental congenital disorders stemming from mutations in the RAS/MAPK pathway, present a unique opportunity to delve into the intricacies of complex neurological disorders. Afflicting approximately one in a thousand newborns, RASopathies manifest as abnormalities across multiple organ systems, with a pronounced impact on the central and peripheral nervous system. In the pursuit of understanding RASopathies’ neurobiology and establishing phenotype–genotype relationships, in vivo non-mammalian models have emerged as indispensable tools. Species such as *Danio rerio*, *Drosophila melanogaster*, *Caenorhabditis elegans*, *Xenopus* species and *Gallus gallus* embryos have proven to be invaluable in shedding light on the intricate pathways implicated in RASopathies. Despite some inherent weaknesses, these genetic models offer distinct advantages over traditional rodent models, providing a holistic perspective on complex genetics, multi-organ involvement, and the interplay among various pathway components, offering insights into the pathophysiological aspects of mutations-driven symptoms. This review underscores the value of investigating the genetic basis of RASopathies for unraveling the underlying mechanisms contributing to broader neurological complexities. It also emphasizes the pivotal role of non-mammalian models in serving as a crucial preliminary step for the development of innovative therapeutic strategies.

## 1. Introduction

RASopathies are a group of syndromic disorders primarily caused by germline mutations in genes that encode any of the proteins along the rat sarcoma/mitogen-activated protein kinase (RAS/MAPK) signaling network. The core of this cascade comprises the membrane-bound RAS-GTPase, the protein kinases RAF and MEK, and the transcription factor ERK (extracellular signal-related kinase), which control numerous cellular and physiological processes, including organism development, cell cycle control, cell proliferation and differentiation, cell survival, and death [1,2]. These disorders manifest as multisystem syndromes, including, but not limited to, neurofibromatosis type 1 (NF1), Noonan syndrome (NS), Legius syndrome (LS), Costello syndrome (CS), cardio–facio–cutaneous (CFC) syndrome and SYNGAP1 encephalopathy (SE) [3]. Most RASopathies are associated with an autosomal dominant mode of inheritance, one exception being Noonan syndrome, where recessive phenotypes caused by mutations in *LZTR1* and *SPRED2* have been described [4,5]. Although each RASopathy syndrome is individually rare, collectively, this family of congenital disorders is one of the largest in the world, affecting approximately 1 in 1000 individuals [6]. Patients affected by these disorders have common clinical features such as short stature, dysmorphic facial features, cardiac structural and functional defects, and lymphatic dysfunction [7]. The central and peripheral nervous systems of patients are also frequently and severely affected, as evidenced by structural malformations of the brain, such as macrocephaly [8], neurocognitive deficits [9], and intellectual disability, as well as an increased risk of tumorigenesis [10]. The symptoms of RASopathies can either present at birth or become apparent later in life. Regrettably, finding effective targeted therapies remains a challenge, and available treatment options are currently scarce or non-existent [11]. It is therefore essential to better define the genetic, molecular and cellular etiologies of these complex congenital diseases to identify effective treatments. The investigation of RASopathies also offers profound insights into the intricate landscape of complex neurological disorders, enriching our comprehension of the molecular mechanisms dictating neurological health. This scientific endeavor contributes fundamentally to the evolving landscape of precision medicine, forging new pathways for the diagnosis and treatment of a spectrum of neurodevelopmental challenges.

Previous works based on in vivo modelling of RASopathies highlight the relevance of developing animal models for the study of these complex diseases [7,12]. Additional studies addressing neurological disorders linked to RASopathies emphasize the involvement of the RAS/MAPK pathway in human neurodevelopmental processes. These investigations explore the impacts of mutations within pathway components on both human subjects and mice [9,13]. In this context, our emphasis lies in elucidating recent advancements in our understanding of the molecular and cellular underpinnings of neurological symptoms in RASopathies, with a special focus on genetically modified non-mammalian animal models. The zebrafish *Danio rerio*, the fly *Drosophila melanogaster*, the worm *Caenorhabditis elegans*, the frogs *Xenopus laevis* and *Xenopus tropicalis* and the chick *Gallus gallus* stand as valuable models for studying the effects of genetic factors on the RAS/MAPK pathway. These models provide unique advantages for studying genetic modifiers and dissecting interactions within the RAS/MAPK pathway while adhering to the “Three Rs” principles—reduction, refinement and replacement—which are aimed at minimizing the number of animals used, refining experimental procedures for animal welfare, and replacing animals with alternative methods whenever possible. Additionally, these models can be effective in capturing some of the complexity and variability seen in the clinical presentations of these conditions. Nevertheless, it is essential to recognize the constraints inherent in these models and to back up observations by using validation in mammalian and human cellular models.

## 2. Neurological Manifestations of RASopathies

The RAS/MAPK pathway is involved in the control of processes of embryonic and postnatal development, such as cell specification and axon growth, and also in the control of adult plasticity of the central and peripheral nervous systems [14,15]. Consequently, genetic mutations associated with RASopathies give rise to multiple neurological impairments [9]: (i) structural intracranial anomalies like Chiari I malformation [16], syringomyelia, cerebral vascular anomalies, benign external hydrocephalus, craniosynostosis and posterior fossa anomalies [17]; (ii) neuropathies or headaches; (iii) seizures [18]; and (iv) diverse cognitive deficits, such as psychomotor delay [19] and cognitive abnormalities [20]. As in other neurodevelopmental disorders, these cognitive deficits consist in language delays [21], deficits in learning ability and memory [20], and social impairments that are partly reminiscent of autism spectrum disorders [22]. All these neurological disorders tend to be lifelong and compromise the well-being of patients and their families. Furthermore, individuals with RASopathies face a heightened susceptibility to both benign and malignant nervous system tumors, such as low-grade gliomas in NS and Chiari 1 malformations in CS [8], an increased rhabdomyosarcoma risk [23], as well as other risks [2,24,25]. RASopathies that manifest with neurological disorders include NF1, LS, NS, CS, CFC syndrome and SE (Table 1). Further details of the neurological manifestations present in each disorder are shown in the following.

Neurofibromatosis type 1 (NF1) is a dominant autosomal disorder caused by mutations in the *NF1* gene [26]. These mutations render NF1-derived neurofibromin protein unable to promote the intrinsic GTPase activity of RAS to hydrolyze RAS-bound GTP to GDP, ultimately activating the RAS signaling pathway [27]. NF1 occurs in approximately one out of every 3200 people, and symptoms normally begin in newborns and infants [28]. Neurological symptoms may be different from person to person, may differ in number and can range from mild to severe (Table 1). Neurological abnormality is very frequently found in NF1 patients, including neurological symptoms and disorders such as hydrocephalus, macrocephaly, cerebrovascular events, neuropathy, seizures, epilepsy, and headache [18]. Patients may have cognitive defects, including mild intellectual disability, which is characterized by an intelligence quotient (IQ) ranging from 50 to 69, memory impairment, neurological speech impairment and specific learning disabilities, such as reading or writing, coordination, self-control or attention, that interfere with the ability to learn [29,30,31]. Behavioral problems may include attention deficits, hyperactivity and impulsivity, thus fulfilling the diagnostic criteria for attention-deficit/hyperactivity disorder (ADHD), as well as an increased incidence of autism spectrum disorder (ASD) [32,33]. NF1 individuals may develop nervous system defects including cerebellar ataxia, glaucoma, meningioma and neurofibromas. Synaptic plasticity alterations have also been detected in adult NF1 patients [34,35]. Finally, they have heightened risk of malignant tumors of the central nervous system (optic-pathway glioma, astrocytoma, and tumors in the brain, on the cranial nerves, or involving the spinal cord) and malignant peripheral nerve sheath tumors (MPNST) [25,36,37,38]. 

Legius syndrome (LS) is a dominant autosomal disorder, caused by heterozygous inactivating mutations in *SPRED1* [39], which negatively regulates the RAS-mediated activation of BRAF, CRAF and neurofibromin [40,41]. The estimated birth prevalence of this disorder is not known, but it is supposed to be very rare, and the age of onset can vary, ranging from newborn to childhood, with a milder phenotype, compared with NF1 patients. Symptoms may include, in rare cases, learning impairments, attention problems, ADHD, abnormal brain imaging, Chiari type 1 malformation, intellectual impairment, speech difficulties and seizures [42] (Table 1). Occasional symptoms include short attention span, whereas frequent neurological symptoms include various affective, behavioral, cognitive and perceptual abnormalities, as well as specific learning disabilities (reading or writing, coordination, or attention, not related to a global deficiency of intelligence), hyperactivity and macrocephaly [43]. While development of tumors is common in NF1, they are rare in LS, with only a few cases reported in the literature [39,44,45]. These findings suggest that *SPRED1* mutations may not confer the same tumorigenic risk as *NF1* mutations, although further research is needed to fully understand the relationship between *SPRED1* and tumorigenesis.

Noonan syndrome (NS) is a dominant autosomal disorder caused by heterozygous mutations in one of several RAS/MAPK pathway genes, although rare autosomal recessive forms have been described [4,46]. In all, 50% of NS cases are caused by mutation in *PTPN11***,** 10% by mutation in *SOS1*, 10% by mutation in *RIT1*, 5% by mutation in *RAF1*, and an additional 5% of cases are caused by mutations in *KRAS* (Table S1). The remaining 20% of cases are caused by rarer mutations in up to 10 different genes (Table S1). NS occurs in approximately one in one thousand to one in twenty-five hundred individuals, with onset ranging from prenatal stages up to 11 years, and symptoms vary in number and severity among patients (Table 1). More than one-third of patients have neurocognitive delay, including lower IQ, ADHD, language problems (dysarthric speech, neurological speech impairment, and sensorineural hearing impairment), delayed verbal recall, and visual recognition memory deficits [47,48]. Social and emotional problems are also seen [47], as well as ASDs [49]. LTP-like synaptic plasticity induced by transcranial magnetic stimulation (TMS) is impaired in Noonan syndrome patients, suggesting underlying synaptic plasticity defects [34]. Other neurological features include high forehead, an intractable neuropathic pain linked to generalized or proximal nerve hypertrophy in the peripheral nerve system in adult patients [50], and an increased risk of developing neurological malignancies, such as low-grade glioma [51].

Costello syndrome (CS) is a RASopathy caused by heterozygous gain-of-function germline mutations in *HRAS* (Table S2), typically in the G12 position of HRAS (p.G12S variant) [52]. Age of onset can vary, ranging from prenatal to newborns. Individuals with CS typically present craniofacial abnormalities such as cerebral cortical atrophy, ventriculomegaly or hydrocephalus in the brain, macrocephaly, and Chiari malformation or demyelination of the basal ganglia [53]. They may also experience seizures and have an increased risk of developing benign or malignant tumors [54]. Cognitive impairment, including mild-to-moderate grade intellectual disability, and affected learning and memory processes are also common features [55] (Table 1). Studies using TMS protocols have found enhanced synaptic plasticity in CS patients, although these studies are from very small cohorts [34,56,57]. 

Cardio–facio–cutaneous syndrome (CFC) syndrome is a dominant genetic disorder caused by alterations in one of four genes: *BRAF* (~75%), *MEK1/2* (~25%) and *KRAS*, which occurs in few individuals, although some other genes might be also associated with CFC syndrome [58] (Table S2). CFC syndrome is a very rare condition whose incidence is unknown, and its age of onset can vary, ranging from prenatal to newborns. Hypotonia, motor delay, speech delay and intellectual disability, and learning disability can be considered the main neurologic features of this syndrome, although it is also characterized by the presence of macrocephaly and brain structural malformations, among other symptoms [58] (Table 1). The presence of ASD is also high in CFC syndrome patients, supporting the importance of the RAS/MAPK pathway in the etiology of ASD [49]. Findings on magnetic resonance imaging have included prominent Virchow–Robin spaces, abnormal myelination and structural anomalies [53]. 

SYNGAP1 encephalopathy (SE) is caused by mutations in the *SYNGAP1* gene. The *SYNGAP1* gene, identified as a causative factor for autosomal dominant intellectual disability type 5 [59] and classified as a RASopathy protein, primarily impacts the central nervous system, functioning as a neuronal RAS GTPase-activating protein (GAP) with roles in the regulation of excitatory plasticity [60,61]. SYNGAP1 loss-of-function mutations have been reported in patients with intellectual disability, schizophrenia, seizures and epilepsy, with patients exhibiting ASD as well [61,62] (Table 1). In particular, large-scale genetic studies utilizing exome sequencing have now related mutations in *SYNGAP1* to increased risk of both ASD [63] and schizophrenia [64]. Moreover, there is evidence of how decreased *SYNGAP1* expression in human neurons precipitates alterations in dendritic and synaptic maturation, resulting in enlarged neurons, heightened excitatory synapse density and earlier onset of synaptic activity [65]. This evidence collectively highlights the role of *SYNGAP1* in neurological processes.

## 3. Neuro-Phenotypic Complexity in RASopathies with Insights from Non-Mammalian Models

Establishing a genotype–phenotype relationship in RASopathies is challenging, given the substantial degree of clinical variability and incomplete penetrance of the associated phenotypes. For instance, mutations occurring in neural progenitor cells during neurogenesis can result in diverse tissue effects within the same person [66,67], and recent studies have demonstrated that particular mutations associated with each disorder disrupt central nervous system (CNS) development in a mutation-specific manner [13]. Non-mammalian in vivo models have proven particularly valuable for validating the impact of RASopathy genes on neurodevelopmental processes reliant on the RAS/MAPK signaling pathway [12,68,69]. Techniques to modulate RAS-MAPK signaling activity include clustered regularly interspaced short palindromic repeats (CRISPR), transcription activator-like effector nucleases (TALENs) or transposon-based methods in Zebrafish and *Drosophila* [12], RNA interference (RNAi) [70], CRISPR-Cas9 gene editing [68] and behavioral assays [71] in *C. elegans*, CRISPR and TALEN in *Xenopus* [72], and in ovo electroporation of expression vectors in chick embryos [73]. The employment of each technique depends on the final goal of the study. For example, overexpression techniques such as mRNA injection and transposon-based methods are desired to mimic a specific phenotype observed in humans or to assay for the activity of the mutant protein in vivo. However, if the aim is to accurately model a disease in heterozygous individuals, it is more effective to introduce mutations at the endogenous locus by using techniques such as TALENs or CRISPR [12]. Bearing this in mind, these models are adept at conducting phenotypic analyses across various biological scales, spanning from the molecular to the tissue level.

*Danio rerio*, the zebrafish, has emerged as a robust non-mammalian model for research due to its high fecundity and time- and cost-efficient genetic manipulation and real-time high-resolution imaging. Zebrafish embryos develop rapidly and have transparent bodies, making it easy to observe developmental defects and organ dysfunction. Several techniques can be employed to validate the consequences of RASopathy genes in neurodevelopmental processes reliant on MAPK signaling in zebrafish, including behavioral assays [74], high-content screening [75], and determining the major/minor axis ratio of embryos [5]. Also, various functional assays have been used for gene discovery, including in vitro luciferase assays and in vivo zebrafish modelling [76,77,78,79]. These techniques have greatly increased the ability to investigate the impact of certain mutations and how these lesions impact disease phenotype, positioning the zebrafish as a powerful in vivo tool for modeling neurological syndromes [80]. In addition, the zebrafish platform supports medium- to high-throughput preclinical drug screening to identify compounds that may represent novel treatment paradigms or even prevent cancer evolution [81]. In fact, zebrafish is emerging as a time- and cost-effective cancer avatar model to assess tumor phenotype and drug responses. For instance, zebrafish patient-derived xenografts (zPDX) may be suitable for providing precision cancer-medicine pipelines [82]. 

**Figure 1:**
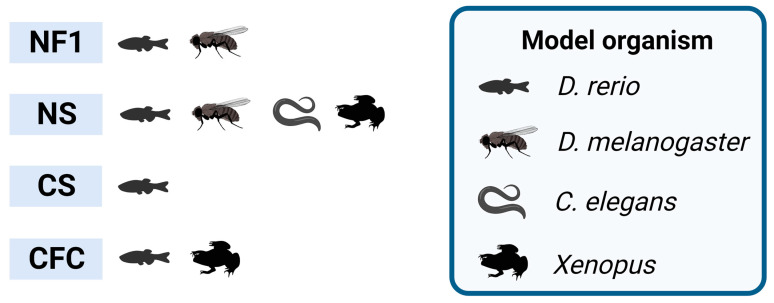
RASopathies explored in non-mammalian models. Non-mammalian models employed in neuroscience research, highlighting the studied syndromes exclusively based on the relevant RASopathy genes. NF1 stands for neurofibromatosis type 1; NS stands for Noonan syndrome; CS stands for Costello syndrome; CFC stands for cardio–facio–cutaneous syndrome. Created with BioRender.com.

Nonetheless zebrafish has been used for the study of NF1, NS, CS and CFC syndromes (Figure 1). Zebrafish has proven to be an effective in vivo model for investigating NF1, with successful knockout of *nf1a* and *nf1b* paralogues displaying macrocephaly and increased oligodendrocyte progenitor cell (OPC) migration within the spinal cord, and defects in myelin structure formation lead to decreased myelination in these mutants [83] (Table S2). Additionally, *nf1* mutants crossed with a p53 null background develop high-grade gliomas and MPNSTs, similar to what is observed in NF1 patients [83]. Also, *NF1* variants associated with cognitive dysfunction have been linked to aberrant cAMP signaling [84]. Memory formation and recall have been tested in *nf1* mutants, and deficits were improved by treatment with chemical stimulants for the cAMP pathway [85]. The over-expression of platelet-derived growth factor receptor alpha (PDGFRA) has been linked to malignant transformation of MPNSTs [86], and over-expression of PDGFRA in a *nf1a*^+/−^; *nf1b*^−/−^; *p53*^m/m^ background accelerated the onset of MPNSTs [87]. Other studied neurological defects include macro- and microcephaly [88], various developmental abnormalities, [76] and tumors [84].

As with *NF1*, the zebrafish genome contains two *PTPN11* genes (*ptpn11a* and *ptpn11b*), encoding Shp2a and Shp2b (Table S1), two proteins with a similar catalytic activity, although only Shp2a is indispensable during zebrafish development [79]. NS-associated neurological features, including shorter body axis length [79,89], craniofacial defects [90], and impaired long-term memory [91], can be attributed to the dynamic regulation of cell movement during zebrafish gastrulation and epiboly [92,93]. E-cadherin turnover, as well as ERK signaling, contribute to these processes. Noonan-associated *PTPN11* and *NRAS* mutants can alter coordinated convergent-extension cell movements and result in oblong embryos with abnormal axis ratios [12,94]. Additionally, introducing *ptpn11* variants associated to NS with multiple lentigines (NSML) in zebrafish embryos can increase the major/minor axis ratio [89] and cause craniofacial dysmorphia in adult fish [79]. Shp2^D61G^ mutant zebrafish can display neurological NS traits, including craniofacial defects, which vary in severity among individuals [95]. The introduction of gain-of-function mutations in RIT1 into zebrafish embryos also causes NS and demonstrates a biological effect similar to mutations in other RASopathy-related genes [76]. The biochemical relationship between the RASopathy proteins KRAS, RIT1 and LZTR1 was tested in zebrafish and fruit flies; all LZTR1 orthologs preferentially interacted with RIT1 orthologs, indicating that this interaction is also conserved in less-complex model organisms [96]. Homozygosity of three different *SPRED2* variants can cause developmental delay, intellectual disability, cardiac defects, short stature, skeletal anomalies, and a typical facial gestalt as major features linked to a recessive phenotype evocative of NS [5] (Table S2). Furthermore, activating *RRAS2* mutations can cause NS, and several zebrafish models were generated to study this; larvae overexpressing RRAS2^G24_G26dup^ or RRAS2^Q72H^ variants, but not wild-type RRAS2, showed craniofacial defects and macrocephaly [97], and RRAS2^Q72L^ caused severe developmental impairments and craniofacial defects, whereas RRAS2^F75C^ resulted in no aberrant in vitro or in vivo phenotypes [97] (Table S2). 

Zebrafish embryos have been employed to probe craniofacial anomalies associated with NS, a condition linked to *A2ML1* variants detected in NS patients (Table S2). In this context, morphometric analysis of *A2ML1* mutant-expressing embryos revealed substantial head broadening and facial blunting [77]. On the other hand, A2ML1 mutations showed no influence on zebrafish development [98]. Notably, since RAC1 activates PAK, which assists in the MEK/ERK pathway via phosphorylation of c-RAF and MEK1 [99], RAC1 variants implicated in developmental delay during neuronal maturation exhibited dominant-negative properties causing microcephaly, diminished neuronal proliferation, and cerebellar anomalies when overexpressed in zebrafish [88]. Intriguingly, the RAC1^P29S^ variant induced RASopathy-like manifestations in zebrafish akin to activated BRAF and KRAS, mitigated by PAK and MEK inhibitors [100]. Furthermore, a RABL3^S36*^ mutant linked to hereditary pancreatic cancer led to RASopathy features in a zebrafish model, with adult homozygous mutants displaying indicative swimming defects [101]. Expression of activated BRAF, KRAS, or RAC1 prompted pronounced body axis disruption and heightened ERK and PAK activity, contrasting with wild-type BRAF’s inert impact [100]. These findings collectively underscore the potency of zebrafish models in elucidating molecular and developmental mechanisms underlying diverse genetic disorders.

Transgenic zebrafish that ubiquitously expresses the constituently active HRAS^G12V^ in the germline display phenotypes consistent with CS: shortened body length, craniofacial dysmorphia and oncogene-induced senescence in the brain [102]. Overexpression of HRAS^G12V^ led to a 22% mortality rate at 2 dpf and a 60% mortality rate at 5 dpf in zebrafish embryos, with 6% of surviving embryos exhibiting brain hemorrhage at 5 dpf [103]. 

The biological consequences of CFC syndrome that have been associated with *BRAF* mutations have been studied in several non-mammalian models, including zebrafish. Expression of the common kinase-activating variant in CFC syndrome BRAF^Q257R^ in zebrafish embryos increased the major/minor axis ratio [104]. Also, overexpression of BRAF^V600E^ before gastrulation in zebrafish embryos caused severe embryonic malignancy with truncated posterior structure and compromised forebrain, while later induction led to craniofacial deformities resembling CFC syndrome [105] (Table S2). Activating mutations in MEK1 have different strengths, which are correlated with the severity of the phenotypes observed in human patients affected by these mutations [106]. Further, spatiotemporal resolution was used in zebrafish to discover that intrinsically active variants of MEK can both increase and reduce the levels of pathway activation in vivo [107].

*Drosophila melanogaster* has been a powerful genetic system with which to decipher fundamental cell and developmental biology questions, including the discovery of components of the RAS/MAPK pathway [108]. *Drosophila* presents many interesting attributes such as its short life-cycle, small size, high fecundity and a relatively compact genome [109]. Furthermore, more than half of *Drosophila* genes have orthologs in the human [110] and its complex brain and behavioral repertoire make it a particularly useful model for understanding neurobiology [111,112]. Results from this model organism may provide valuable insights into how pathogenic variants promote specific traits in humans, as well as enable tailored therapeutic approaches to treat them. *Drosophila* has been used for the study of NF1 and NS (Figure 1). Early work in *Drosophila* identified the ortholog of ERK as the terminal kinases of the RAS/ERK signaling cascade [113]. Later on, *Drosophila* RASopathy models were generated, generally emulating RAS gain-of-function phenotypes found in NS patients, by mutating the *Drosophila* ortholog of human *PTPN11*, *corkscrew* (*csw*) [90,91], and *SOS2* [114]. *Drosophila* wing vein formation and eye development have been settled as excellent in vivo readouts for RAS signaling [115,116]. For example, expression of *PTPN11* variants associated in NS-like in *Drosophila* results in ectopic wing vein and rough eye phenotypes [117], and NS-associated mutations in *RIT1* also result in ectopic wing veins [76]. Studies have shown that the loss of neurofibromin affects the synaptic function of neurons, which is associated with a decrease in neurotransmitter release, changes in synaptic plasticity and abnormal activation of signaling pathways involved in neuronal development and function [118,119]. Although the *NF1* mutations affect both types of memory, the GAP domain that regulates RAS affects only long-term memory [118,119], while the C-terminal domain reduces the immediate memory through regulation of adenyl cyclase [120]. Interestingly, this memory impairment could be rescued by expression of a human *NF1* transgene [118,119]. Other studies also support the proposition that *Drosophila* lacking the *NF1* homolog exhibit specific learning and memory deficits similar to those of many children with NF1 [121,122]. Expression of *Dsor1*^Y130C^, the MEK1 ortholog in *Drosophila*, leads to larval cuticle deficits and ectopic wing veins [106] (Table S2), and intrinsically active variants of MEK can produce either an increase or a decrease in pathway activation levels in vivo, as previously reported for zebrafish [107]. Aberrant RAS/MAPK signaling in the eye leads to small, rough eyes; aberrant signaling in the wing leads to ectopic wing development [7]. *Drosophila* Lztr1 preferentially regulates Ric rather than Ras levels, as seen with the corresponding mammalian orthologs [96]. However, a previous study showed that flies expressing RNA interference constructs against *Lztr1* displayed minor defects in wing vein patterning [123]. Divergent outcomes between these two studies may arise from the incomplete knockdown and off-target effects in the RNA interference study by Bigenzahn et al. (2018), compared to the complete loss of function achieved in the null *Lztr1* flies of the Cuevas-Navarro et al. (2022) study, which was potentially compounded by differences in experimental conditions. 

Studies in *Drosophila* also identified novel modifiers (Dap160 and CCKLR-17D1) that implicate synaptic defects in the dNf1 growth deficiency [124]. This and other studies also have implicated the neuronal-specific RTK Anaplastic Lymphoma Kinase (ALK) as an upstream activator of RAS signaling in neurons [124,125]. Altering ALK activity in these animal models partially ameliorates NF1 cognitive deficits, suggesting that it represents a potential therapeutic target. More recently, 13 *Drosophila* RASopathy models expressing commonly observed RASopathy variants were generated and used to explore fly phenotypes, altered cellular signaling networks, and responses to drugs [126] (Table S2). Using up to 33 *Drosophila* models, it was planned to provide a broad overview of how RASopathy variants alter the signaling network in different tissues [11]. 

*Caenorhabditis elegans* stands as a potent model to unveil the pathogenic implications of RASopathy mutations. *C. elegans* is a small nematode worm that has become a promising model organism for studying neurological diseases due to its simple and well-characterized nervous system. It has a transparent body, enabling researchers to study the effects of genetic and environmental factors on its development, physiology, and behavior. With only 302 neurons and ~7000 synapses, it has the ability to recapitulate key aspects of neurological function, and to unravel complex signaling pathways involved in human disease systems [127,128]. The genome of *C. elegans* contains homologs of two-thirds of all human disease genes, including many related to neurological disorders [129]. In recent years, *C. elegans* has also been used for high-throughput drug screening, further highlighting its potential as a model organism for studying neurological diseases [130]. *C. elegans* has been used for the study of NS (Figure 1). Mutations in the *MAPK1* gene cause the multivulva (Muv) phenotype, which is consistent with aberrant RAS/MAPK pathway activity. Up to five (p.Ile74Asn, p.His80Tyr, p.Ala174Val, p.Asp318Gly and p.Pro323Arg) de novo MAPK1 variants found in patients with a neurodevelopmental disease within the RASopathy phenotypic spectrum were expressed in *C. elegans*, and all of them were found to cause the Muv phenotype [68] (Table S2). Similarly, the SHOC2^S2G^ variant, which is involved in neurodevelopmental disorders including NS [131], was found to introduce a N-myristoylation site, resulting in an impaired translocation to the nucleus upon growth factor stimulation, and engendered protruding vulva, a phenotype also associated with an aberrant signaling in the RAS/MAPK pathway [132] (Table S2). In addition, expression of the RRAS^G39dup^, identified in a panel of NS patients, ortholog in *C. elegans* enhanced RAS signaling and engendered protruding vulva, as well as decreased egg-laying efficiency and accumulation of larvae inside the mother, supporting the proposition of the gain-of-function role of the mutation in RAS-1 function [133] (Table S2). 

*Xenopus* has been used as a model organism in the study of embryology, cell biology, genetics, physiology, toxicology and disease, among other fields [134]. This amphibian has several advantages as a model system, including its external development, easy genetic manipulation, and availability of a variety of techniques for the study of neural development and function, along with the balance between mesoderm formation and the levels of MAPK [135]. Furthermore, one of the main advantages of its use is that the disruptions of development caused by RASopathy-associated mutations can be effectively modeled in *Xenopus*, presenting phenotypes that are highly reminiscent of the symptoms of patients and thus allowing the shedding of light into the mechanisms by which these diseases develop [69]. *Xenopus* has been used for the study of NS and CFC syndrome (Figure 1). Induction of a dominant-negative SHP2 in *Xenopus* blocks mesoderm formation by impairing ERK signaling, leading to arrest of gastrulation [136,137]. Also, *Xenopus* has been used to study the effects of mutations in the RAS/MAPK pathway on neural crest (NC) cell migration, a key process in the development of the peripheral nervous system [138]. *Xenopus* embryos expressing a dominant negative FGFR4 showed reduced NC gene expression at mid-neurula stages [14]. When a CFC syndrome germline variant in 14-3-3ζ (*YWHAZ*) was expressed in *Xenopus tropicalis*, it caused an increase in BRAF and RAF1 binding, ERK phosphorylation, and a decrease in body length [139]. Furthermore, recent studies in *Xenopus* have revealed the role of the RAS/MAPK pathway in the development of the visual system [140]. Overall, the use of *Xenopus* as a non-mammalian model system has proven to be a valuable tool in the study of the neurobiology of RASopathies. 

## 4. Other Potential Models: Chick Embryos

Leveraging chick embryos as a model organism provides a valuable avenue for unraveling intricate molecular mechanisms underpinning neurological disorders. Aberrant RAS signaling activation in chick embryos notably induces shifts in axon guidance [141]. Additionally, mutations in RAS/MAPK components prompt alterations in cortical neuron migration and morphological changes [142]. Intriguingly, investigations have illuminated the pivotal role of PI3K as an anti-apoptotic transducer in neuroblasts and neurons, while also governing cellular migration within the neuroepithelium through Rho GTPases. Recent advancements showcase that an overactivation of ERK1/2 in the trunk neural tube, mediated by the constitutive active form of MEK1 (MEK1ca), drives shifts in the transcriptional profile of developing spinal cord cells. MEK1ca-transfected cells relinquish neuronal identity, expressing potential oncogenes like AQP1, highlighting MEK1’s promise as an in vivo model in uncovering mechanisms fostering neoplasia and malignancy in neural-origin ERK-induced tumorigenesis [73]. This model is notably advantageous for examining early embryonic development, a pivotal phase for brain maturation [143]. 

The chicken embryo, with a resemblance to the human embryo’s frontonasal mass surpassing that of the mouse, offers an invaluable platform for craniofacial research, bolstered by the capacity for replicative accumulation [144]. Techniques, including RNAi, morpholinos, promoter-driven DNA constructs for gain of function, ex ovo early embryo electroporation, and in ovo electroporation, stand at our disposal to investigate neurodevelopmental gene involvement in chick embryos [145]. A distinctive advantage lies in the chicken embryo’s capacity for comprehensive observation, enabling the association of genes or signals with phenotypes. These studies, uncovering the effects of RAS signaling modulation on chick embryo development, underscore the species’ potential in delving into the etiology of neurological disorders associated with RASopathies [144].

## 5. Limitations of Using Non-Mammalian Models

While non-mammalian models offer significant advantages for studying various diseases, including RASopathies, they are not without limitations that warrant consideration. In general terms, there are limitations when studying some of the most fundamental aspects of development, genetics, pathology, and disease mechanisms unique to humans in animal models. These drawbacks are even higher if the study is focused on disorders that affect the brain, where the most significant differences between humans and animal models have been found. Neuronal subtype complexity and human-specific aspects of gene expression and regulation are some of the key differences that limit the ability of animal models to recapitulate human brain development and to be used in identifying the underlying cellular and molecular mechanisms [146]. Another limitation to take in account is the difference between human and animal models in response to drugs, as pharmacokinetics and pharmacodynamics vary not only between species but also between individuals from the same species [147]. Also, it is well known that mammalian models are evolutionary closer to humans and thus are more similar to them than are non-mammalian models [148].

Zebrafish has a comparatively lower brain complexity when compared to humans and mammals, and this, coupled with the field’s relatively early stage, presents challenges. Also the variability in responses due to a low inbreeding rate can complicate data analysis [149]. *Drosophila* models mainly rely on human disease-causing gene overexpression in fly eyes, which lack the complexity and significant anatomical differences seen in the human brain [150]. In the case of *C. elegans*, its simplicity in terms of replicating human neural connections and cell interactions, which is critical for understanding the pathogenesis of neurodegenerative diseases, represents a limitation [129]. The yolk content within *Xenopus* embryos can hinder signal detection, and deep live imaging within early embryos is constrained in conventional imaging methods. Additionally, the longer generation time and reproduction cycle can impact experimental frequency [151]. Susceptibility to high doses of potentially lethal chemicals and the influences of poorly understood factors like breeder genetics, age, egg size, and incubation conditions are limitations in using chick embryos [152,153].

## 6. Effective Neurological Treatments and RASopathy Inhibitors Used in Non-Mammalian Models

**Figure 2:**
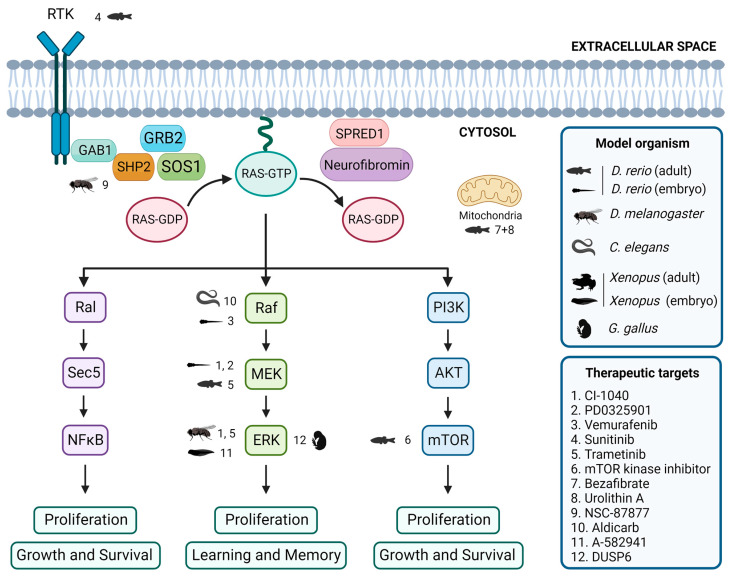
Evaluation of inhibitors and drugs for RASopathies in non-mammalian models. Overview of inhibitors and drugs tested in non-mammalian models, showing the associations between tested drugs, target proteins, and the corresponding non-mammalian models. Created with BioRender.com.

Currently, there are no definitive cures for RASopathies. However, ongoing research focuses on the exploration of the RAS/MAPK signaling pathway, aiming to develop pharmaceutical interventions that may effectively alleviate neurological symptoms. However, the complexity of the signaling pathway and the diversity of the symptoms associated with RASopathies make drug development challenging. Still, there are several treatments and therapies available, based on the medical issues of each patient [7,11,19,154]. Neuropharmacology is a promising approach for the treatment of neurological symptoms in RASopathies, and studies using non-mammalian model organisms may help to identify potential therapeutic targets (Figure 2). 

In zebrafish embryos, studies have shown that expression of BRAF^Q257R^ increased the major/minor axis ratio, a phenotype that was prevented by treatment with the MEK inhibitors CI-1040 and PD0325901 [104,155]. Similarly, the CFC-syndrome-like phenotypes associated with ectopic expression of BRAF^V600E^ were profoundly ameliorated by simultaneous treatment with vemurafenib [105]. Furthermore, in the zebrafish model of NF1, treatment with sunitinib, a RTK inhibitor, effectively arrested the progression of transplanted MPNSTs, and combination treatment with both sunitinib and trametinib, a MEK inhibitor, enhanced this therapeutic effect [87]. Additionally, the study of the effects of various drugs on the zebrafish models of RASopathies has allowed the identification of targets of the RAS/MAPK pathway and the investigation of the mechanisms underlying the pathogeneses of RASopathies [69]. DNA Topo I and mTOR kinase inhibitors were identified as the most effective single agents in eliminating MPNST cells while avoiding excessive toxicity [156]. Treatment with a combination of bezafibrate and urolithin A provided a synergistic effect at a mitochondrial level, rescuing the genetic developmental defects in the CS zebrafish model [103].

The *Drosophila* model has been instrumental in identifying potential therapeutic targets for RASopathy-associated symptoms [126,157]. For example, preclinical studies using *Drosophila* have shown that inhibitors of the Ras/MAPK pathway, such as SHP2 inhibitor NSC-87877 [91] and MEK inhibitors CI-1040 [104] and trametinib [87], have potential therapeutic value. Studies in flies have also shown that activating the cAMP pathway can rescue neurofibromin deficiency [158]. In addition, the gene *nemy* has been identified as a potential therapeutic target for the treatment of cognitive impairment [159]. 

In *C. elegans*, expression of activated LIN-45/Raf in head acetylcholine neurons is sufficient to cause a waveform phenotype and hypersensitivity to the acetylcholinesterase inhibitor aldicarb, similar to an activated heterotrimeric G protein (Gq) mutant, suggesting that the ERK/MAPK pathway modulates the output of Gq-Rho signaling to control locomotion [160]. Furthermore, although not directly studied in RASopathy models, the GABAergic signaling pathway [161] and acetylcholine system [162] have been identified as potential therapeutic targets for treating cognitive impairment. 

Several studies using *Xenopus* have identified potential therapeutic targets for treating neurological symptoms that may be present in patients affected by RASopathies. One study found that the use of A-582941, an agonist of the α7 nicotinic acetylcholine receptor (α7 nAChR), can lead to broad-spectrum efficacy at doses that enhance ERK1/2 and cAMP response element-binding protein (CREB) activation, and may represent a mechanism that offers potential for the improvement of cognitive deficits [163]. In another study, the effects of a 14-3-3ζ variant found in CFC syndrome patients were investigated, and the results suggested that the inhibition of the RAS/MAPK pathway could potentially be a therapeutic approach used to ameliorate CFC syndrome symptoms [139]. 

In chick embryos, only a limited number of studies have focused on the identification of key signaling pathways and targets that may be relevant in this context. One study investigated the effects of arsenic on NC cells in chick embryos and found that it induced gross abnormalities in craniofacial development and neural tube defects [164]. Inhibition of ERK1/2 activity by overexpressing the ERK1/2 phosphatase DUSP6 reduced the expression of the NC genes, although the way by which ERK1/2 signaling promotes NC induction is still unclear [14].

## 7. Discussion

The spectrum of neurological symptoms associated with RASopathies is underpinned by a myriad of factors, including the progressive nature of the disease, genetic modifiers, the intricate interplay of RASopathy-related gene mutations, and the relationships between tumor suppressors and neural tumors. Acknowledging the evolving nature of their symptoms over an individual’s lifespan and comprehending the genetic nuances that drive symptom diversity are paramount to a holistic understanding of the disease’s clinical landscape. Furthermore, a more comprehensive exploration of neural tumors within the context of RASopathies would provide a well-rounded depiction of the neurological intricacies that patients encounter. Importantly, delving into the intricacies of neural symptoms within the context of RASopathies not only contributes to a more thorough understanding of the associated neurological complexities, but also has broader implications, potentially shedding light on fundamental aspects of neurological diseases that are more complex.

The use of non-mammalian model organisms, such as zebrafish, *Drosophila*, *C. elegans*, *Xenopus* and chick embryos, has been instrumental in advancing our understanding of RASopathies and developing new neuropharmacological approaches. No single model can fully replicate human diseases, and further research is required to bridge the gap between these models and clinical settings. Non-mammalian model organisms provide a simplified system for studying the RAS/MAPK signaling pathway and the underlying mechanisms of neuroprotection and neurotoxicity, which can be challenging to study in mammalian models. Based on the current literature, it seems that some of the less common RASopathies, such as NSML and CFC syndrome, have not been studied as extensively as some of the more common RASopathies like NF1 and NS. There are still several common RASopathy variants identified in humans that have yet to be modeled in non-mammalian models. For example, the *PTPN11* mutation A72T is common in NS but has not been studied in any non-mammalian model. Similarly, the RAF1 mutation G266E, which is also commonly found in NS, has not been studied in any non-mammalian models. In addition, some mutations that have been studied in non-mammalian models have not been identified as common variants in humans. For example, the RAF1 mutation S259A has been studied in zebrafish but is not a common variant in humans [165]. It is important to note that the study of RASopathies is an ongoing and constantly evolving field, and new variants are constantly being identified and studied. So, while some variants may not have been modeled yet, they may be studied in the future as new research is conducted.

Non-mammalian models have significantly contributed to the quest for effective treatments in the realm of RASopathies. Studies in *C. elegans* have underscored the intricate relationship between the ERK/MAPK pathway and locomotion, indirectly hinting at potential intervention points. *Zebrafish* models have shown promise in assessing RASopathy inhibitors, with MEK inhibitors and RTK inhibitors demonstrating potential therapeutic value. *Drosophila* has played a pivotal role in identifying therapeutic targets for RASopathy-associated cognitive deficits, including inhibitors of the RAS/MAPK pathway and the activation of the cAMP pathway. *Xenopus* models have unveiled potential targets for the amelioration of cognitive deficits in RASopathies, and although chick embryos are relatively less explored in this context, they have begun shedding light on the impact of environmental factors and ERK1/2 signaling. The chick embryo model emerges as an indispensable asset used for uncovering the underlying mechanisms of neurological disorders within the spectrum of RASopathies. Its utility extends to the identification of new neuroprotective compounds and a heightened comprehension of the RAS-MAPK pathway’s role in neurodevelopment. Significantly, the potential of chick embryos in drug discovery for RASopathies is underscored, demonstrating their transformative capacity in advancing therapeutic avenues. These non-mammalian models offer diverse advantages, from cost-effectiveness to the ability to study genetic modifiers and pathway interactions, collectively aiding in the quest for effective treatments in the complex landscape of neurobiology.

Current research suggests a dynamic interplay between non-mammalian, mouse and iPSC models in RASopathy research. *Zebrafish* and *C. elegans* models have identified key signaling pathways and potential therapeutic targets, which have then been validated and further characterized in mice. Subsequently, iPSC models can be used to refine these findings in a human context, considering individual patients’ variations. This multi-model approach strengthens the translational potential of research findings and increases the likelihood of successful therapeutic development. While non-mammalian models offer valuable starting points, mice and iPSCs play critical roles in validating and translating their findings towards clinical application. The future lies in a collaborative approach, leveraging the strengths of each model system to bridge the gap between basic research and effective therapies for RASopathies and other complex diseases. Additionally, advanced computational tools are emerging to integrate data from diverse models, providing a more comprehensive understanding of RASopathies.

While non-mammalian models have been invaluable in understanding RASopathies and developing therapeutic approaches, the future may hold even more revolutionary solutions. The emergence of powerful technologies like artificial intelligence (AI) has the potential to redefine the way we think about and develop therapies, potentially even questioning the need for traditional model systems altogether. These systems could identify intricate patterns and connections that might be missed by traditional research methods, leading to the discovery of novel therapeutic targets and personalized treatment strategies tailored to individual patients’ unique genetic makeup. AI could also be used to simulate complex biological processes, including disease progression and drug interactions, with a level of detail and accuracy that surpasses current model systems. However, it is crucial to remember that it is a tool, not a replacement for biological understanding, and it still needs further development to accurately predict or stimulate the outcome of a disease and drug interaction. Model systems, especially those incorporating human-derived cells or organoids, will still play a vital role in validating AI-generated predictions and testing the safety and efficacy of potential therapies in a more biological context. The convergence of AI and other emerging technologies will undoubtedly reshape the landscape of therapeutic discovery, offering exciting possibilities for the future of medicine.

## Figures and Tables

**Table 1:** Neurological phenotypes in patients with RASopathies; the most common neurological features observed in individuals affected by RASopathies. The ✔ symbol denotes the presence of a specific phenotype within the respective RASopathy, while the X symbol indicates its absence. The category “Increased risk of tumors” encompasses both benign and malignant tumors. NF1 stands for neurofibromatosis type 1; LS stands for Legius syndrome; NS stands for Noonan syndrome; CS stands for Costello syndrome; CFC stands for cardio–facio–cutaneous syndrome; SE stands for SYNGAP1 encephalopathy; ADHD stands for attention-deficit/hyperactivity disorder; ASD stands for autism spectrum disorder.

Neurological Feature	NF1	LS	NS	CS	CFC	SE
Intellectual disability	✔	✔	✔	✔	✔	✔
Cranial, facial or brain malformations	✔	✔	✔	✔	✔	X
Learning disability	✔	✔	✔	✔	✔	X
Seizures/Epilepsy	✔	✔	X	✔	X	✔
ASD	✔	X	✔	X	✔	✔
ADHD/Hyperactivity	✔	✔	✔	X	X	X
Increased risk of tumors	✔	X	✔	✔	X	X
Social impairments	X	X	✔	X	X	X
Hypotonia	X	X	X	X	✔	X
Schizophrenia	X	X	X	X	X	✔

## Supplementary Materials

The following supporting information can be downloaded at: https://www.mdpi.com/article/10.3390/biomedicines12040841/s1, Table S1: Conservation of the RASopathy genes in non-mammalian model organisms. This table illustrates the percentage of identity between genes associated with RASopathies and their orthologs in non-mammalian models, focusing exclusively on those genes and models mentioned in the text. In genes associated with Noonan syndrome (NS), asterisks are used to indicate an additional connection with Noonan Syndrome with Multiple Lentigines (NSML, denoted by *) or syndromes resembling Noonan syndrome (**). Data have been obtained from DIOPT Ortholog Finder (accessed on 31 August 2023, https://www.flyrnai.org/cgi-bin/DRSC_orthologs.pl) and Ensembl Bio Mart (accessed on 31 August 2023, https://www.ensembl.org/info/data/biomart/index.html); Table S2: RASopathy-associated mutations studied in non-mammalian model organisms. Collation of investigated RASopathy-associated mutations in non-mammalian models emphasized in this review, along with observed related features. In genes related to Noonan syndrome (NS), double asterisks (**) signify an additional correlation between the mutations and Noonan-like syndrome.
