# Supplementary material for: Non-Mammalian Models for Understanding Neurological Defects in RASopathies
Source: Biomedicines. 2024 Apr 10;12(4):841. doi: 10.3390/biomedicines12040841 (PMC11048513; doi:10.3390/biomedicines12040841)
Supplement: Supplementary file 1 [file biomedicines-12-00841-s001.zip › Table S1. Conservation of the RASopathy genes in non-mammalian model organisms.pptx]

## Slide 1
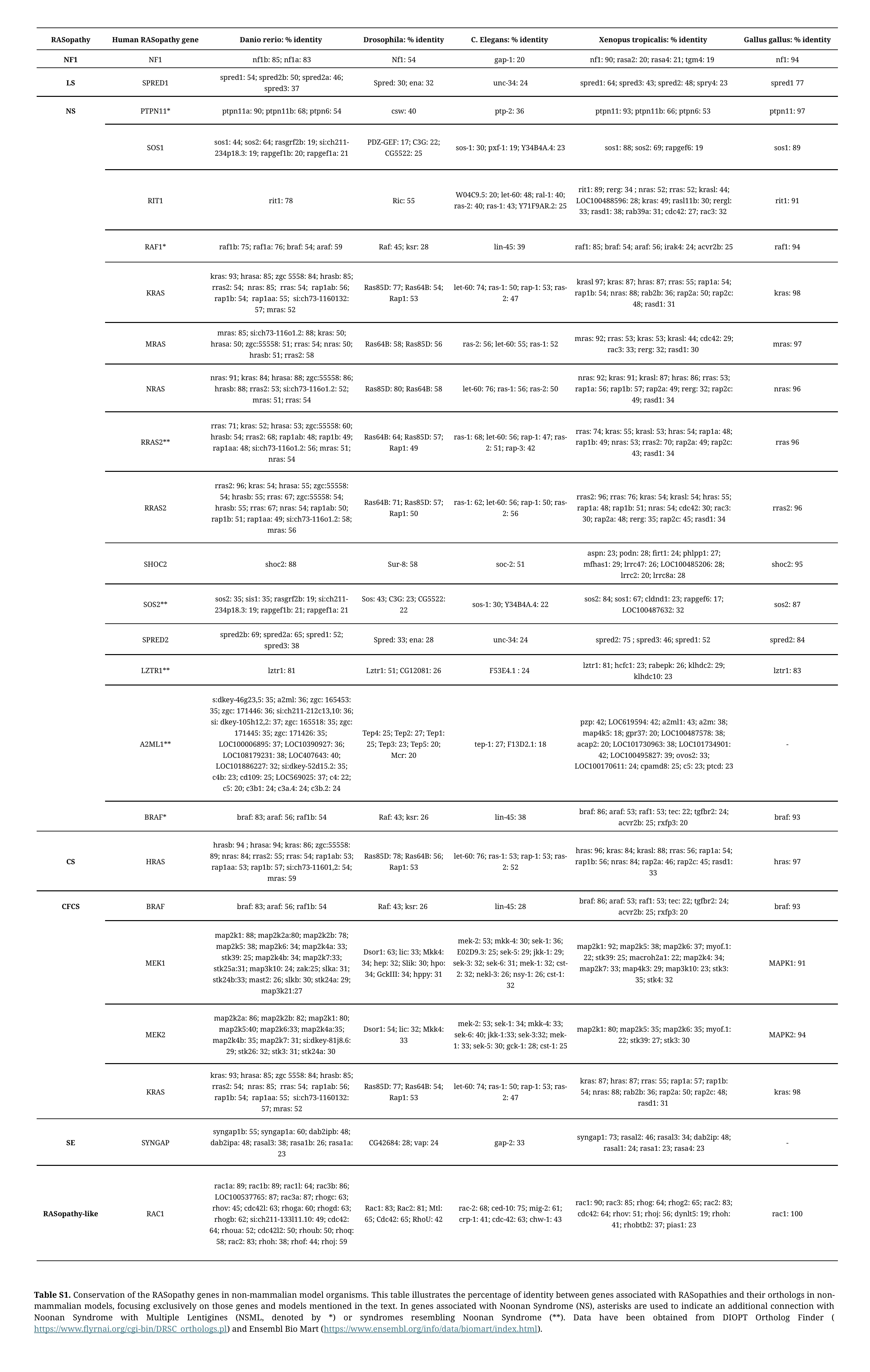

| RASopathy | Human RASopathy gene | Danio rerio: % identity | Drosophila: % identity | C. Elegans: % identity | Xenopus tropicalis: % identity | Gallus gallus: % identity |
| --- | --- | --- | --- | --- | --- | --- |
| NF1 | NF1 | nf1b: 85; nf1a: 83 | Nf1: 54 | gap-1: 20 | nf1: 90; rasa2: 20; rasa4: 21; tgm4: 19 | nf1: 94 |
| LS | SPRED1 | spred1: 54; spred2b: 50; spred2a: 46; spred3: 37 | Spred: 30; ena: 32 | unc-34: 24 | spred1: 64; spred3: 43; spred2: 48; spry4: 23 | spred1 77 |
| NS | PTPN11\* | ptpn11a: 90; ptpn11b: 68; ptpn6: 54 | csw: 40 | ptp-2: 36 | ptpn11: 93; ptpn11b: 66; ptpn6: 53 | ptpn11: 97 |
| | SOS1 | sos1: 44; sos2: 64; rasgrf2b: 19; si:ch211-234p18.3: 19; rapgef1b: 20; rapgef1a: 21 | PDZ-GEF: 17; C3G: 22; CG5522: 25 | sos-1: 30; pxf-1: 19; Y34B4A.4: 23 | sos1: 88; sos2: 69; rapgef6: 19 | sos1: 89 |
| | RIT1 | rit1: 78 | Ric: 55 | W04C9.5: 20; let-60: 48; ral-1: 40; ras-2: 40; ras-1: 43; Y71F9AR.2: 25 | rit1: 89; rerg: 34 ; nras: 52; rras: 52; krasl: 44; LOC100488596: 28; kras: 49; rasl11b: 30; rergl: 33; rasd1: 38; rab39a: 31; cdc42: 27; rac3: 32 | rit1: 91 |
| | RAF1\* | raf1b: 75; raf1a: 76; braf: 54; araf: 59 | Raf: 45; ksr: 28 | lin-45: 39 | raf1: 85; braf: 54; araf: 56; irak4: 24; acvr2b: 25 | raf1: 94 |
| | KRAS | kras: 93; hrasa: 85; zgc 5558: 84; hrasb: 85; rras2: 54; nras: 85; rras: 54; rap1ab: 56; rap1b: 54; rap1aa: 55; si:ch73-1160132: 57; mras: 52 | Ras85D: 77; Ras64B: 54; Rap1: 53 | let-60: 74; ras-1: 50; rap-1: 53; ras-2: 47 | krasl 97; kras: 87; hras: 87; rras: 55; rap1a: 54; rap1b: 54; nras: 88; rab2b: 36; rap2a: 50; rap2c: 48; rasd1: 31 | kras: 98 |
| | MRAS | mras: 85; si:ch73-116o1.2: 88; kras: 50; hrasa: 50; zgc:55558: 51; rras: 54; nras: 50; hrasb: 51; rras2: 58 | Ras64B: 58; Ras85D: 56 | ras-2: 56; let-60: 55; ras-1: 52 | mras: 92; rras: 53; kras: 53; krasl: 44; cdc42: 29; rac3: 33; rerg: 32; rasd1: 30 | mras: 97 |
| | NRAS | nras: 91; kras: 84; hrasa: 88; zgc:55558: 86; hrasb: 88; rras2: 53; si:ch73-116o1.2: 52; mras: 51; rras: 54 | Ras85D: 80; Ras64B: 58 | let-60: 76; ras-1: 56; ras-2: 50 | nras: 92; kras: 91; krasl: 87; hras: 86; rras: 53; rap1a: 56; rap1b: 57; rap2a: 49; rerg: 32; rap2c: 49; rasd1: 34 | nras: 96 |
| | RRAS2\*\* | rras: 71; kras: 52; hrasa: 53; zgc:55558: 60; hrasb: 54; rras2: 68; rap1ab: 48; rap1b: 49; rap1aa: 48; si:ch73-116o1.2: 56; mras: 51; nras: 54 | Ras64B: 64; Ras85D: 57; Rap1: 49 | ras-1: 68; let-60: 56; rap-1: 47; ras-2: 51; rap-3: 42 | rras: 74; kras: 55; krasl: 53; hras: 54; rap1a: 48; rap1b: 49; nras: 53; rras2: 70; rap2a: 49; rap2c: 43; rasd1: 34 | rras 96 |
| | RRAS2 | rras2: 96; kras: 54; hrasa: 55; zgc:55558: 54; hrasb: 55; rras: 67; zgc:55558: 54; hrasb: 55; rras: 67; nras: 54; rap1ab: 50; rap1b: 51; rap1aa: 49; si:ch73-116o1.2: 58; mras: 56 | Ras64B: 71; Ras85D: 57; Rap1: 50 | ras-1: 62; let-60: 56; rap-1: 50; ras-2: 56 | rras2: 96; rras: 76; kras: 54; krasl: 54; hras: 55; rap1a: 48; rap1b: 51; nras: 54; cdc42: 30; rac3: 30; rap2a: 48; rerg: 35; rap2c: 45; rasd1: 34 | rras2: 96 |
| | SHOC2 | shoc2: 88 | Sur-8: 58 | soc-2: 51 | aspn: 23; podn: 28; firt1: 24; phlpp1: 27; mfhas1: 29; lrrc47: 26; LOC100485206: 28; lrrc2: 20; lrrc8a: 28 | shoc2: 95 |
| | SOS2\*\* | sos2: 35; sis1: 35; rasgrf2b: 19; si:ch211-234p18.3: 19; rapgef1b: 21; rapgef1a: 21 | Sos: 43; C3G: 23; CG5522: 22 | sos-1: 30; Y34B4A.4: 22 | sos2: 84; sos1: 67; cldnd1: 23; rapgef6: 17; LOC100487632: 32 | sos2: 87 |
| | SPRED2 | spred2b: 69; spred2a: 65; spred1: 52; spred3: 38 | Spred: 33; ena: 28 | unc-34: 24 | spred2: 75 ; spred3: 46; spred1: 52 | spred2: 84 |
| | LZTR1\*\* | lztr1: 81 | Lztr1: 51; CG12081: 26 | F53E4.1 : 24 | lztr1: 81; hcfc1: 23; rabepk: 26; klhdc2: 29; klhdc10: 23 | lztr1: 83 |
| | A2ML1\*\* | s:dkey-46g23,5: 35; a2ml: 36; zgc: 165453: 35; zgc: 171446: 36; si:ch211-212c13,10: 36; si: dkey-105h12,2: 37; zgc: 165518: 35; zgc: 171445: 35; zgc: 171426: 35; LOC100006895: 37; LOC10390927: 36; LOC108179231: 38; LOC407643: 40; LOC101886227: 32; si:dkey-52d15.2: 35; c4b: 23; cd109: 25; LOC569025: 37; c4: 22; c5: 20; c3b1: 24; c3a.4: 24; c3b.2: 24 | Tep4: 25; Tep2: 27; Tep1: 25; Tep3: 23; Tep5: 20; Mcr: 20 | tep-1: 27; F13D2.1: 18 | pzp: 42; LOC619594: 42; a2ml1: 43; a2m: 38; map4k5: 18; gpr37: 20; LOC100487578: 38; acap2: 20; LOC101730963: 38; LOC101734901: 42; LOC100495827: 39; ovos2: 33; LOC100170611: 24; cpamd8: 25; c5: 23; ptcd: 23 | - |
| | BRAF\* | braf: 83; araf: 56; raf1b: 54 | Raf: 43; ksr: 26 | lin-45: 38 | braf: 86; araf: 53; raf1: 53; tec: 22; tgfbr2: 24; acvr2b: 25; rxfp3: 20 | braf: 93 |
| CS | HRAS | hrasb: 94 ; hrasa: 94; kras: 86; zgc:55558: 89; nras: 84; rras2: 55; rras: 54; rap1ab: 53; rap1aa: 53; rap1b: 57; si:ch73-11601,2: 54; mras: 59 | Ras85D: 78; Ras64B: 56; Rap1: 53 | let-60: 76; ras-1: 53; rap-1: 53; ras-2: 52 | hras: 96; kras: 84; krasl: 88; rras: 56; rap1a: 54; rap1b: 56; nras: 84; rap2a: 46; rap2c: 45; rasd1: 33 | hras: 97 |
| CFCS | BRAF | braf: 83; araf: 56; raf1b: 54 | Raf: 43; ksr: 26 | lin-45: 28 | braf: 86; araf: 53; raf1: 53; tec: 22; tgfbr2: 24; acvr2b: 25; rxfp3: 20 | braf: 93 |
| | MEK1 | map2k1: 88; map2k2a:80; map2k2b: 78; map2k5: 38; map2k6: 34; map2k4a: 33; stk39: 25; map2k4b: 34; map2k7:33; stk25a:31; map3k10: 24; zak:25; slka: 31; stk24b:33; mast2: 26; slkb: 30; stk24a: 29; map3k21:27 | Dsor1: 63; lic: 33; Mkk4: 34; hep: 32; Slik: 30; hpo: 34; GckIII: 34; hppy: 31 | mek-2: 53; mkk-4: 30; sek-1: 36; E02D9.3: 25; sek-5: 29; jkk-1: 29; sek-3: 32; sek-6: 31; mek-1: 32; cst-2: 32; nekl-3: 26; nsy-1: 26; cst-1: 32 | map2k1: 92; map2k5: 38; map2k6: 37; myof.1: 22; stk39: 25; macroh2a1: 22; map2k4: 34; map2k7: 33; map4k3: 29; map3k10: 23; stk3: 35; stk4: 32 | MAPK1: 91 |
| | MEK2 | map2k2a: 86; map2k2b: 82; map2k1: 80; map2k5:40; map2k6:33; map2k4a:35; map2k4b: 35; map2k7: 31; si:dkey-81j8.6: 29; stk26: 32; stk3: 31; stk24a: 30 | Dsor1: 54; lic: 32; Mkk4: 33 | mek-2: 53; sek-1: 34; mkk-4: 33; sek-6: 40; jkk-1:33; sek-3:32; mek-1: 33; sek-5: 30; gck-1: 28; cst-1: 25 | map2k1: 80; map2k5: 35; map2k6: 35; myof.1: 22; stk39: 27; stk3: 30 | MAPK2: 94 |
| | KRAS | kras: 93; hrasa: 85; zgc 5558: 84; hrasb: 85; rras2: 54; nras: 85; rras: 54; rap1ab: 56; rap1b: 54; rap1aa: 55; si:ch73-1160132: 57; mras: 52 | Ras85D: 77; Ras64B: 54; Rap1: 53 | let-60: 74; ras-1: 50; rap-1: 53; ras-2: 47 | kras: 87; hras: 87; rras: 55; rap1a: 57; rap1b: 54; nras: 88; rab2b: 36; rap2a: 50; rap2c: 48; rasd1: 31 | kras: 98 |
| SE | SYNGAP | syngap1b: 55; syngap1a: 60; dab2ipb: 48; dab2ipa: 48; rasal3: 38; rasa1b: 26; rasa1a: 23 | CG42684: 28; vap: 24 | gap-2: 33 | syngap1: 73; rasal2: 46; rasal3: 34; dab2ip: 48; rasal1: 24; rasa1: 23; rasa4: 23 | - |
| RASopathy-like | RAC1 | rac1a: 89; rac1b: 89; rac1l: 64; rac3b: 86; LOC100537765: 87; rac3a: 87; rhogc: 63; rhov: 45; cdc42l: 63; rhoga: 60; rhogd: 63; rhogb: 62; si:ch211-133l11.10: 49; cdc42: 64; rhoua: 52; cdc42l2: 50; rhoub: 50; rhoq: 58; rac2: 83; rhoh: 38; rhof: 44; rhoj: 59 | Rac1: 83; Rac2: 81; Mtl: 65; Cdc42: 65; RhoU: 42 | rac-2: 68; ced-10: 75; mig-2: 61; crp-1: 41; cdc-42: 63; chw-1: 43 | rac1: 90; rac3: 85; rhog: 64; rhog2: 65; rac2: 83; cdc42: 64; rhov: 51; rhoj: 56; dynlt5: 19; rhoh: 41; rhobtb2: 37; pias1: 23 | rac1: 100 |
Table S1. Conservation of the RASopathy genes in non-mammalian model organisms. This table illustrates the percentage of identity between genes associated with RASopathies and their orthologs in non-mammalian models, focusing exclusively on those genes and models mentioned in the text. In genes associated with Noonan Syndrome (NS), asterisks are used to indicate an additional connection with Noonan Syndrome with Multiple Lentigines (NSML, denoted by *) or syndromes resembling Noonan Syndrome (**). Data have been obtained from DIOPT Ortholog Finder (https://www.flyrnai.org/cgi-bin/DRSC_orthologs.pl) and Ensembl Bio Mart (https://www.ensembl.org/info/data/biomart/index.html).
