# Supplementary material for: Non-Mammalian Models for Understanding Neurological Defects in RASopathies
Source: Biomedicines. 2024 Apr 10;12(4):841. doi: 10.3390/biomedicines12040841 (PMC11048513; doi:10.3390/biomedicines12040841)
Supplement: Supplementary file 1 [file biomedicines-12-00841-s001.zip › Table S2. RASopathy-associated mutations studied in non-mammalian model organisms.pdf]

RASopathy	Model organism	RASopathy gene	Mutation	Features	Reference
NF1	*Danio rerio*	NF1	nf1a-/-<br>nf1b-/-	Macrocephaly and increased OPC migration within the spinal cord	81
NS	*Danio rerio*	*PTPN11*	Shp2 p.D61G	Craniofacial defects	93
		RRAS2	RRAS2 p.G24_26dup<br>RRAS2 p.Q72H	Craniofacial defects and macrocephaly	95
			RRAS2 p.Q72L	Craniofacial defects and developmental impairment	
			RRAS2 p.F75C	No aberrant in vitro or in vivo phenotypes	
		*A2ML1***	A2ML1 p.R592L<br>A2ML1 p.R802L<br>A2ML1 p.R802H	Craniofacial defects that are characteristic of NS, including a broad head	75
		SPRED2	SPRED2 p.R63*<br>SPRED2 p.L100P<br>SPRED2 p.L381Hfs*95	NS evocative phenotypes, such as developmental delay and intellectual disability	4
	Drosophila	*PTPN11*	Shp2 p.D61G<br>Shp2 p.Y279C<br>Shp2 p.N308D<br>Shp2 p.R498W<br>Shp2 p.Q510P<br>Shp2 p.Q510E	Altered cellular signaling networks	119
		BRAF	BRAF1 p.Q257R<br>BRAF p.W531C	Altered cellular signaling networks	119
		KRAS	KRAS p.G12D	Altered cellular signaling networks	119
		HRAS	HRAS p.G12S	Altered cellular signaling networks	119
		RAF1	RAF1 p.S257L<br>RAF1 p.D486G<br>RAF1 p.L613V	Altered cellular signaling networks	119
	C. elegans	*MAPK1*	MAPK1 p.I74N<br>MAPK1 p.H80Y<br>MAPK1 p.A174V<br>MAPK1 p.D318G<br>MAPK1 p.P323R	Muv phenotype, consistent with aberrant RAS/MAPK pathway activity	66
		SHOC2	SHOC2 p.S2G	Aberrant RAS/MAPK signaling	125
		RRAS	RRAS p.G39dup	Enhanced RAS/MAPK signaling	126
CS	*Danio rerio*	*HRAS*	HRAS p.G12V	CS phenotypes such as craniofacial dysmorphia and oncogene-induced senescence in the brain	99
CFC	*Danio rerio*	*BRAF*	BRAF p.Q257R	Increased major:minor axis ratio	101
			BRAF p.V600E	Early induction: truncated posterior structure and compromised forebrain. At a later stage, craniofacial deformities	102
	Drosophila	*MEK1*	Dsor1 p.Y130C	Larval cuticle deficits and ectopic wing veins	103
RASopathy-like	*Danio rerio*	*RAC1*	RAC1 p.P29S	Rasopathy-like phenotype	97
		RABL3	RABL3 p.S36*	Rasopathy-like phenotype	98

Table S2. RASopathy-associated mutations studied in non-mammalian model organisms. Collation of investigated RASopathy-associated mutations in non-mammalian models emphasized in this review, along with observed related features. In genes related to Noonan Syndrome (NS), double asterisks (**) signify an additional correlation between the mutations and Noonan-like syndrome.
